# Immune Crosstalk Between Lymph Nodes and Breast Carcinomas, With a Focus on B Cells

**DOI:** 10.3389/fmolb.2021.673051

**Published:** 2021-05-28

**Authors:** Elena Alberts, Isobelle Wall, Dinis Pedro Calado, Anita Grigoriadis

**Affiliations:** ^1^Faculty of Life Sciences and Medicine, Cancer Bioinformatics, School of Cancer & Pharmaceutical Sciences, King’s College London, London, United Kingdom; ^2^Breast Cancer Now Unit, School of Cancer and Pharmaceutical Sciences, King’s College London, London, United Kingdom; ^3^Faculty of Life Sciences and Medicine, School of Cancer & Pharmaceutical Sciences, King’s College London, London, United Kingdom; ^4^Immunity and Cancer Laboratory, The Francis Crick Institute, London, United Kingdom

**Keywords:** lymph node, TILs, tertiary lymphoid structures, germinal centers, breast cancer

## Abstract

Lymph nodes (LNs) are highly organized secondary lymphoid organs, and reflective of immune responses to infection, injuries, or the presence of cancer. Extensive molecular and morphological analyses of immune and stromal features in tumors and LNs of breast cancer patients have revealed novel patterns indicative of disease progression. Within LNs, there are dynamic structures called germinal centers (GCs), that act as the immunological hubs for B cell development and generation of affinity matured memory B and antibody-producing plasma cells. Acting as a bridge between systemic and local immunity, associations are observed between the frequency of GCs within cancer-free LNs, the levels of stromal tumor infiltrating lymphocytes, and cancer progression. Scattered throughout the tumor microenvironment (TME) or aggregated in clusters forming tertiary lymphoid structures (TLS), the occurrence of tumor infiltrating B cells (TIL-Bs) has been linked mostly to superior disease trajectories in solid cancers. Recent TIL-Bs profiling studies have revealed a plethora of different TIL-B populations, their functional roles, and whether they are derived from GC reactions in the LN, and/or locally from GC-like structures within the TME remains to be investigated. However, parallels between the immunogenic nature of LNs as a pre-metastatic niche, TIL-B populations within the TME, and the presence of TLS will help to decipher local and widespread TIL-Bs responses and their influence on cancer progression to the lymphatics. Therapies that enhance TIL-Bs responses in the LN GC and/or in GC-like structures in the TME are thus emerging management strategies for breast and other cancer patients.

## Highlights

- Morphological alterations in cancer-free and cancer-involved axillary lymph nodes hold predictive prognostic information for breast cancer patients.
- Tumor infiltrating B cells (TIL-Bs) can form tertiary lymphoid structures that bear morphological and cellular similarities to germinal centers in lymph nodes.
- TIL-Bs at the primary tumor site and the formation of tertiary lymphoid structures are mostly associated with superior disease trajectories in breast and other solid cancers.

- Deep single-cell profiling has revealed a plethora of markers and new TIL-B populations, and for many their functional role remains to be determined.
- Exploring the responses of TIL-Bs at the primary tumor, in peritumoral germinal center-like structures and in lymph nodes will inform the development of novel therapies.

## Introduction

Lymph nodes (LNs) are secondary lymphoid organs that act as a platform to facilitate antigen dispersal and promote interactions between immune cell subsets. In response to disease, inflammatory chemokines and cytokines mediate the recruitment of lymphocytes and antigen-presenting cells that access the LNs via lymphatic vessel-mediated lymph drainage. Passing through the subcapsular sinus, these lymph-borne solutes disseminate into the cortex where B cells, intrafollicular T cells, and dendritic cells are arranged in a highly size-restricted manner, before moving through conduits to reach the T cell zone of the LN paracortex. Depending on the nature of the stimuli, these compartments can expand or diminish to generate an optimal B cell response. Eventually, lymphocytes exit the LNs via efferent lymphatic vessels, and the LNs return to a naïve, resting state (Willard-Mack, 2006).

Despite serving as transportation channels essential to an effective immune response, the lymphatics also act as corridors for cancerous cells to pass through into the LNs (Stacker et al., 2014; Ji, 2016). Typically presenting as the initial seeding site outside of the primary tumor, the presence of metastatic growth within LNs has been associated with both shortened disease-free survival and a heightened risk of developing metastases in distant organs. Therefore, the incidence of cancer cells within the LNs, the number of metastatic LNs, and the occurrence of extra nodal extension have formed essential assessment parameters for routine pathological diagnosis of several cancers, including breast cancer.

Historically, axillary LN clearance was undertaken for all patients with invasive breast cancer. Today, the standard treatment of care for patients with clinically and radiologically negative nodes prior to surgery is surgical resection of only the nodes adjacent to and draining from the tumor bed, the so-called sentinel LNs. It is also becoming more common for breast cancer patients to receive neoadjuvant chemotherapy (NACT), which presents new challenges for the assessment of the LNs. Treatment-induced fibrosis and reactive changes can obscure the local environment and prevent an accurate diagnosis of LN metastasis. Thus, exploring the spatial, cellular, and molecular alterations in LNs occurring due to immune surveillance of nearby tumor growth, as well as from NACT or immunotherapy will expand our understanding of the immune crosstalk between LNs and breast carcinomas.

Germinal centers (GC) are immunological sites in the LN within which B cell receptor (BCR) affinity maturation occurs to generate long-lived memory B and plasma cells (Victora and Nussenzweig, 2012; Nakagawa et al., 2021). Tightly regulated mechanisms within the GC promote targeted responses to pathogens whilst ensuring the elimination of autoreactive clones. B cells retrieve antigen via their BCR from follicular dendritic cells (FDCs) and present that antigen to follicular T helper (Tfh) cells (Victora and Nussenzweig, 2012). GC B cells compete for Tfh derived signals critical for positive selection, which culminates in the upregulation of the transcription factor MYC (Calado et al., 2012; Dominguez-Sola et al., 2012). BCR affinity maturation in GC B cells occurs through iterative rounds of clonal expansion, somatic hypermutation and selection to generate fine-tuned humoral responses (Victora and Nussenzweig, 2012). Owing to their highly organized structures, morphological alterations in GCs indicates fluctuations in their molecular mechanisms and polarization, which ultimately has an impact on the quality and quantity of the resulting memory B and plasma cell populations (Victora et al., 2010; Bannard et al., 2013; Toboso-Navasa et al., 2020). Early autochthonous models of breast cancer showed enlarged GCs in regional LNs with increased levels of lymphoblasts, a sign of active lymphopoieses, and clusters of dividing plasma cells in the vicinity of the GC (Ciocca, 1980).

**FIGURE 1:**
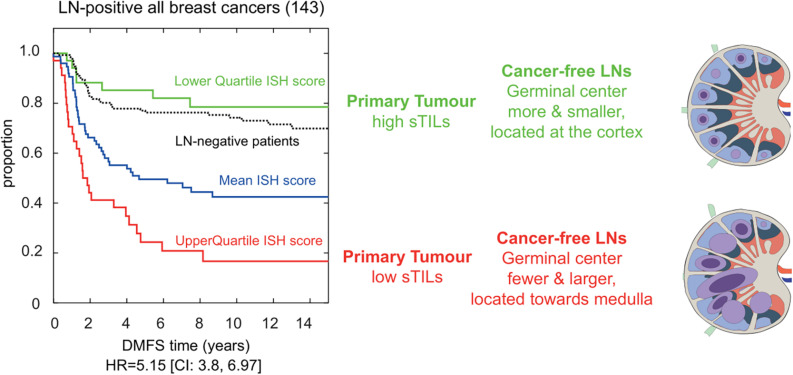
Immune-stroma-histological (ISH) score is risk-predictive of developing distant metastasis in breast cancer patients. Cox proportional hazards regression analysis of LN-positive breast cancer patients (n:143) separated based on their immune and stromal histomorphological features. We developed an ISH-risk score capturing several different immune and stromal features at the primary tumor side, in cancer-free and metastatic lymph nodes. Patients with high scores had shorter time to distant metastasis (red line in Kaplan–Meier graph). Their cancer-free LNs had fewer and larger germinal centers (GCs; blue circles) located toward the center of the node. Cancer-free LN with smaller and more GCs were (blue circles) found in patients with longer time to metastasis (green line in Kaplan–Meier graph). These patients had a better prognosis than LN-negative patients (black lines in Kaplan–Meier). Hazard ratio (HR) and confidence interval (CI) are shown below the graph, modified from Grigoriadis et al. (2018).

Based on extensive H&E assessment of immune and stromal features at primary breast carcinomas and patient-matched axillary LNs, we were the first to report on histological changes in cancer-free LNs carrying additive risk predictive value for developing distant metastasis (Grigoriadis et al., 2018). By incorporating the number and size of GCs in cancer-free LNs, combined with the level of stromal tumor-infiltrating lymphocytes (sTILs) at the primary tumor, the presence of lymphocytic lobulitis, and location of GCs in the involved LN, an immune-stroma-histological (ISH)-risk score was implemented indicative for the risk of developing distant metastasis for breast cancer patients (Figure 1). Amongst the 309 breast cancer patients enriched of triple-negative phenotype and of high histological grade, a group of LN-positive patients with the lowest quartile ISH scores showed a superior outcome even when compared to LN-negative breast cancer patients. In particular, the number of GC formations in cancer-free LNs added valuable information to TILs in triple-negative breast cancer prognostication (Liu et al., 2021). Multiple questions emerge from this clinically relevant association, and further investigations are needed to dissect the mechanisms driving the formation of LN GCs in breast cancer patients and to understand the function of GC derived memory B and plasma cells in this context.

## Tumor Infiltrating B Cell Responses at the Primary Breast Carcinomas

Triple-negative breast cancers (TNBCs), defined by their lack of expression of estrogen receptor, progesterone receptor, and human epidermal growth factor receptor 2 (HER2), are associated with a poorer prognosis and higher rates of distant recurrence compared to receptor-positive breast cancers (Reddy et al., 2018; Yin et al., 2020). Crucially, lymphatic involvement is more prevalent in TNBC and contributes to both local and distant metastasis (Shen et al., 2014; van Roozendaal et al., 2016; Yao et al., 2019). Both TNBC and HER2-positive breast cancers often present with more immune infiltrated tumors compared to other subtypes, and sTIL assessment of these breast carcinomas have been shown to be superior to classical TNM staging when predicting outcome and response to anti-HER2 therapy, chemotherapy, and immunotherapy (Salgado et al., 2015; Ignatiadis et al., 2019; Loi et al., 2019, 2020). In contrast to the longstanding characterization and manipulation of tumor infiltrating T cells (TIL-Ts) for clinical research, the potential of tumor infiltrating B cells, denoted (TIL-Bs), has only recently sparked interest amongst breast cancer researchers. The majority of breast carcinomas present with relatively low levels of TIL-Bs (∼2–3%), yet this is heightened compared to healthy breast tissue (Garaud et al., 2019). A variety of TIL-Bs at multiple stages of differentiation, including naïve, GC-like, memory-like, and plasma cells (Chung et al., 2017; Garaud et al., 2019) have been reported within and around the tumor microenvironment (TME) of breast tumors, with a high percentage of TIL-B exhibiting a memory-like phenotype (Buisseret et al., 2017). Notably, the occurrence of GC-like B cells consistently correlates with numbers of Tfh cells, signifying an active and constantly evolving humoral response (Garaud et al., 2019). Using multiplexed ion beam imaging to analyze spatial information, TIL-B populations were found to be consistently depleted along the tumor border of TNBCs (Keren et al., 2018), however the cause of this relative reduction is unknown. The spatial patterns and colocalizations with other cell types further indicate the involvement of TIL-Bs in humoral immunity, with possible roles in antigen presentation and modulation of other immune populations with relevance to tumor progression. Particularly in TNBC patients, increased levels of TIL in residual disease post-NACT has been associated with a better prognosis (Dieci et al., 2014). Specifically, the presence of TIL-B at primary tumor lesions is shown to be an independent predictor of NACT response (García-Martínez et al., 2014), and the coexistence of TIL-B and PD-L1-positive carcinoma cells is significantly associated with a high pathological complete response rate and overall survival (Arias-Pulido et al., 2018). Treatment-induced B cell lymphopenia, often seen to predominantly deplete class-switched memory B cells, may impact B cell orientated protection against tumor progression (Mellios et al., 2010; Verma et al., 2016; Gustafson et al., 2020). It is important to consider that TIL-Bs are shown to re-populate at a much slower rate than other immune cells after chemotherapy (Verma et al., 2016; Gustafson et al., 2020), as post-treatment profiles of patients with high TIL-B levels identifies a highly responsive group of breast cancer patients (Song et al., 2017).

## Tumor Infiltrating B Cells in Tertiary Lymphoid Structures

In addition to antibody production and antigen presentation, B cells may contribute to peritumoral immunity by associating with T cells to form organized structures known as tertiary lymphoid structures (TLS) (Germain et al., 2015; Shen et al., 2018). In comparison with GCs in LNs, TLSs are non-encapsulated, transient structures induced and maintained in chronically inflamed tissues. The presence of TLS has been correlated with higher TIL-B levels in the peritumoral area and is frequently associated with superior disease free and overall survival (Figenschau et al., 2015; Lee et al., 2016; Sautès-Fridman et al., 2019). Mature TLS bear similar morphological and molecular characteristics to secondary lymphoid follicles, forming a definitive marginal zone, mantle zone and a central GC-like structure in which B cell centroblast, centrocyte subsets, and Tfh cells are found (Garaud et al., 2019). Somatic mutations of the Ig variable domains of TIL-Bs isolated from T/B clusters in ductal carcinomas revealed evidence of local oligoclonal expansion of cells that have previously undergone antigen-driven hypermutation, proliferation, and affinity maturation, much like within a GC (Nzula et al., 2003). To facilitate recruitment, positioning and interactions within GCs, Tfh cells can express the checkpoint molecules ICOS and PD-1 that engage with cognate ICOSL^+^PD-L1^+^ centrocytes (Shi et al., 2018). Although the proportion of PD-1^+^ and PD-L1^+^ TILs is low in breast cancers, much of the PD-1 is primarily expressed on T cells that can also localize within the TLS. This brings to light the impact of PD-1 as a marker of immune activation in contrast to its connotations with immune exhaustion in the context of cancer. Moreover, expression of PD-1 and PD-L1 in TILs is significantly associated with improved clinical outcomes in TNBC and HER2-enriched breast cancers (Solinas et al., 2017; Schmid et al., 2018).

Yet to be explored in breast cancer, detailed phenotypic characterizations of TLS within other solid cancers such as hepatocellular carcinoma or colorectal cancers identified those of an “early stage” and “immature” phenotype lacking a central GC-like structure (Meylan et al., 2020). In patients with non-metastatic colorectal cancer, tumors presenting with more than one mature TLS that harbors active GC-like interactions were associated with a significantly reduced risk of recurrence compared to patients with solely immature TLS (Posch et al., 2017). In contrast, in oral squamous cell carcinoma patients, no differences in overall or recurrence free survival was observed when the density of immature to mature TLS was compared (Li et al., 2020). In lung squamous cell carcinoma, the existence of TLS was the strongest independent factor when patients were untreated, whilst in patients treated with NACT and corticosteroid therapy, the development of mature TLS seemed to be impaired and were not informative of disease progression (Siliòa et al., 2018). In immature TLS, TIL-B might interact more with cancerous cells than with T cells, and one hypothesis is that those TIL-B release factors that dampen the response of other immune cells, in turn hindering the targeting and destruction of tumor cells. Three recent studies provide indirect evidence that immature TLS are associated with low activity of T cells in tumors, whilst mature TLS nurture B cell development (Cabrita et al., 2020; Helmink et al., 2020; Petitprez et al., 2020). The above-listed disease-specific findings highlight the need to explore in depth the formation and the development of immature and mature TLS responses within the context of breast carcinomas, to establish their drivers, their pro/anti-tumor properties, and the potential impact of neoadjuvant treatment on their formation.

## Transcriptional Profiling of the GC Reaction and of GC B Cell Derived Subsets

Tightly controlled transcriptional profiles govern the formation and development of GCs, and potentially by extension TLS. In the LN, GC B cells transit between two functionally distinct compartments, the dark zone (DZ) and light zone (LZ) which represent polarized areas in which gene expression patterns drive somatic hypermutation and selection, respectively (Calado et al., 2012; Dominguez-Sola et al., 2012; Victora and Nussenzweig, 2012). By utilizing single-cell RNA-sequencing (scRNA-seq) on tissue derived from human and mouse, a plethora of transcriptional changes occurring in GCs has been revealed, and with it novel cell populations have been defined (McHeyzer-Williams et al., 2015; Holmes et al., 2020; Kennedy et al., 2020; King et al., 2021; Nakagawa et al., 2021). Whilst the functional roles for some of these populations remain to be determined, the identification of defining markers provides the opportunity to determine their spatial distribution and possible isolation for further study. scRNA-seq experiments have also tentatively identified the gene expression profiles of memory B and plasma cell precursors in the LZ of GCs. The differentiation of LZ B cells toward the plasma cell fate is associated with increased Tfh help that enhances NF-κB signaling, *IRF4*, *XBP1*, *FKBP11*, and *PRDM1* (*BLIMP1*) expression (Klein et al., 2006; Heise et al., 2014; Nutt et al., 2015; Ruer-Laventie et al., 2015; Ise et al., 2018; Holmes et al., 2020). In contrast, memory B cell differentiation from LZ B cells is restricted to positively selected cells, seemingly requires minimal Tfh help and associates with increased *BACH2* and *HHEX1* expression levels (Sidwell and Kallies, 2016; Laidlaw and Cyster, 2020; Laidlaw et al., 2020; Toboso-Navasa et al., 2020; Nakagawa et al., 2021). The transposition of these datasets to TLS in breast cancer, together with the ability to record the temporal, spatial and transcriptional profiles of GCs and TLS may further our understanding of the TIL-B populations within the TME and provide a rationale for their contribution to disease progression.

## Antitumor and Autoantibody Production in Breast Cancer

As a product of the GC response, plasma cells that have undergone somatic hypermutation and affinity maturation are typically long lived and capable of evoking a humoral response for many years (Brynjolfsson et al., 2018). By contrast, those that develop in extrafollicular foci do not undergo somatic hypermutation, are typically short-lived, and secrete a combination of switched or unswitched antibodies (Paus et al., 2006). Comprehensive gene expression studies of TIL-B populations in breast cancer identified IgG-associated gene sets in primary carcinomas indicative of pathological complete response to trastuzumab combination therapies and superior overall survival in TNBC (Perou et al., 1999; Carey et al., 2014; Iglesia et al., 2014). Spatial analysis of such antibody responses revealed that breast lesions with high levels of tumor infiltrating plasma cells present with antibodies in their tumor core, at the invasive margin and within the stromal compartments (Seow et al., 2020). Some of these antibodies bind tumor cells and display a clonal relationship with those present in the axillary LNs, indicative of a systemic response beyond the local TME (Novinger et al., 2015). Supporting a functional role for antibodies in breast cancer, mice deficient for antibody production display a more aggressive disease progression, and the adoptive transfer of IgG secreting plasma cells present in tumor draining LNs limits metastatic spread (Li et al., 2011; Tao et al., 2013; Brynjolfsson et al., 2018; Hollern et al., 2019). However, the antigen specificity of these functionally relevant antibodies is not completely understood. Conversely, the analysis of the IgG and IgA autoantibody repertoire in breast cancer patients revealed that autoantibodies to one or more tumor-associated antigens occurred in most patients. Notably, patients with a higher level of IgG reactivity to breast cancer-associated antigens have significantly shorter recurrence free survival (Garaud et al., 2018). These findings align with studies of spontaneous LN metastasis breast cancer mouse models. Here, the presence of IgG antibodies to a breast cancer antigen promoted tumor progression through the lymphatics (Gu et al., 2019). It remains unclear whether GC reactions contribute to the production of protective and/or tumor promoting antibodies, and the extent to which GC reactions in the context of breast cancer follow the canonical checkpoints that curb self-reactivity in physiology. This knowledge is clinically relevant as it may provide insight for strategies that selectively inhibit the development of tumor promoting antibodies and enhance cancer-protective humoral immunity.

## Immune Tolerance and Regulation

**FIGURE 2:**
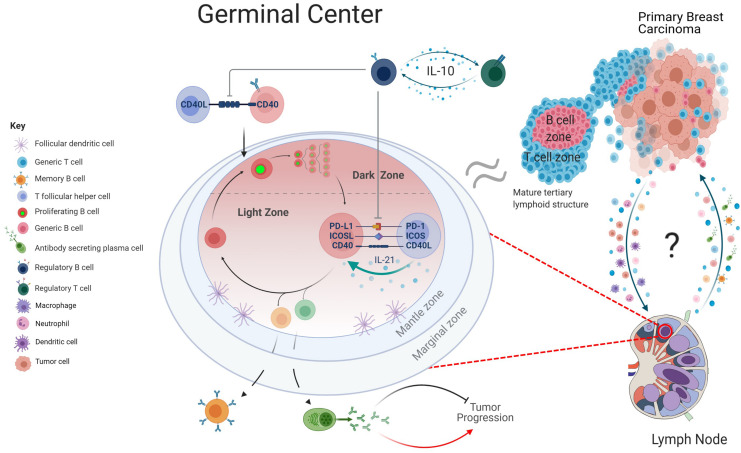
Graphical depiction of GC regulation. Within the lymph nodes (LNs), B cells primed by T cells at the T/B border move into the GC and proliferate rapidly in the dark zone (DZ), before transitioning into the light zone. Here, engagement with T follicular helper cells through checkpoint inhibitor mechanism and cytokines such as IL-21 ensures affinity-based maturation of GC B cells. Upon upregulation of transcriptionally distinct profiles, the B cells differentiate into either a memory B cell, antibody secreting plasma cell or return to the DZ for additional proliferative rounds and hypermutation. Within the context of breast cancer, antibody production has been linked to both the inhibition and promotion of tumor progression. Regulatory B cell subsets can regulate this mechanism by suppressing Tfh mediated activation of B cells and promote an immune tolerant environment through secretion of IL-10. The transition of cancerous cells from the primary tumor to the lymphatics may be accompanied by a multitude of immune cell subsets, including antigen presenting cells. Further communication between LN and tumor and potential migration of B cell subsets, cytokines, and chemokines may be informative of how the GC responses can influence the tumor microenvironment. As the morphology of tertiary lymphoid structures is comparable to that of a B cell follicle, there may be mechanistic parallels that can be drawn between the two structures (created with BioRender.com).

An appropriate immune response relies on a “goldilocks window” of immune checkpoint control; too little regulation promotes the expansion of autoreactive cells, whereas exacerbated expression leads to anergy and exhaustion. Tolerance of GC responses is in part maintained by T follicular regulatory (Tfr) cells, that dampen immune responses by preventing CD28-B7 co-stimulatory interactions through CTLA-4 engagement (Miles and Connick, 2018). Circulating Tfr and Treg cells are enriched in breast cancer patients, particularly in more aggressive cancers (Kohrt et al., 2005; Song et al., 2019; Núñez et al., 2020). This may correlate with the knowledge that Tfr cells potently inhibit antigen-specific antibody responses (Linterman et al., 2011). Tfr and Treg cells can induce an immunosuppressive microenvironment, often through IL-10 production, and promote the expansion of immunosuppressive B cells, so-called Bregs. In a cyclical fashion, IL-10 secreting Bregs support the Treg pool and impede Tfh responses within the GC (Liu et al., 2016; Achour et al., 2017; Song et al., 2019). The number and localization of Bregs in breast carcinomas strongly fluctuates with levels of Tregs, notably in and around B and T cell TIL aggregates (Guan et al., 2016; Ishigami et al., 2019). Supporting a relationship between Bregs and Tregs, a B cell deficient breast cancer model displayed a reduced fraction of Tregs in tumor draining LNs and peritumoral areas (Tadmor et al., 2011). Accumulation of Tregs and Bregs within the cancer-free LNs of breast cancer patients correlates with fewer class-switched B cells in adjacent LNs with cancerous growth, indicative of a possible role in GC suppression (Mehdipour et al., 2016). This synergistic relationship between Bregs and Tregs, their ability to promote a tolerant environment and suppress Tfh cells may influence the efficacy of GC reactions within breast cancer patients (Figure 2). Supporting this hypothesis, IL-10 blockade *in vivo* stimulates IgG production and enhances immune infiltration within the primary breast carcinoma (Zheng et al., 2012; Li and Xia, 2015; Tao et al., 2015).

Bregs express the immune checkpoint molecules PD-1 and PD-L1 which have immunomodulatory functions (Sun et al., 2019). The emerging role of Bregs in promoting immunosuppression in breast cancer may indicate these cells as candidate targets in immune-checkpoint (ICP) blockade therapy. ICP blockades have been shown to promote the proliferation of class-switched memory B cells and enhance antibody production *in vitro* and within the context of chronic infection (Pioli et al., 2000; Velu et al., 2009; Intlekofer and Thompson, 2013). However, a potential effect of immune checkpoint inhibitors on the GC reactions must be considered given that GC B cells themselves express PD-L1, and Tfh cells require many of these immune checkpoint interactions including the PD-1/PD-L1 axis and CTLA-4 for B cell affinity maturation (Good-Jacobson et al., 2010; Hams et al., 2011).

## Conclusion and Future Directions

During the past decade, molecular and cellular parallels between GCs in LNs and TLS formation at the primary tumor suggest a degree of communication via the lymphatics between the sites and revealed a key role for B cells in breast and other cancers. However, we do not understand the processes that render B cell activation able to eradicate neoplastic cells through immunoglobulin-mediated mechanisms versus those leading to chronic B cell activation that potentiates tumor progression. By facilitating an immune crosstalk between LNs, TLS and primary carcinomas, B cells may modulate the dynamic interplay between immune responses and tumor progression. Uncovering the mechanistic drivers that influence specific B cell environments at the tumor side, peritumoral and in the LNs may provide attractive targets, many of which could be incorporated into current immunotherapies to treat both breast and other cancers.

## Author Contributions

EA, IW, DC, and AG wrote a complete draft and first version of the manuscript. All authors contributed to the manuscript revision, read, and approved final version and contributed to the principal layout of the article.

## Conflict of Interest

The authors declare that the research was conducted in the absence of any commercial or financial relationships that could be construed as a potential conflict of interest.
